# Preparation of extracellular matrix of fish swim bladders by decellularization with supercritical carbon dioxide

**DOI:** 10.1186/s40643-022-00621-4

**Published:** 2023-02-21

**Authors:** Yuqing Han, Bingyan Zhang, Jinjin Li, Lian Cen, Ling Zhao, Zhenhao Xi

**Affiliations:** 1 State Key Laboratory of Chemical Engineering, School of Chemical Engineering, East China University of Science and Technology, Shanghai, 200237 China; 2 Shanghai Key Laboratory of Multiphase Materials Chemical Engineering, East China University of Science and Technology, Shanghai, 200237 China

**Keywords:** Supercritical carbon dioxide, Fish swim bladder, Sodium dodecyl sulfate, Decellularization, Interaction energy

## Abstract

Fish swim bladders used to be considered as byproducts or waste in fishery; however, they are potential materials for biological medicine with abundant collagen. In this work, an efficient noncytotoxic decellularization process using sodium dodecyl sulfate (SDS) ternary system assisted with supercritical carbon dioxide (scCO_2_) as the green extraction fluid and ethanol (ET) as the cosolvent has been developed to harvest acellular fish swim bladders (AFSBs). The experimental results show that the tissue treated by SDS assisted with scCO_2_ and ethanol at 37 °C and 25 MPa can be decellularized thoroughly and maintains intact fibers and uniform pore distribution, which resulting in a tensile strength of 5.61 MPa and satisfactory biocompatibility. Meanwhile, the residual SDS content in scCO_2_/SDS/ET ternary system is 0.0122% which is significantly lower than it in scCO_2_/SDS system due to the enhanced mass transfer rate of SDS in tissues by scCO_2_ with ethanol. The synergy between SDS and ethanol can enhance the diffusion coefficient and the solubility of SDS in scCO_2_, which reduced the contact time between SDS and tissues. Meaningfully, the results obtained in this work can not only provide a novel strategy to produce acellular matrix with superior properties, but also offer a further understanding of the decellularization through scCO_2_ extraction processing with the synergy of suitable detergent/cosolvent.

**Graphical Abstract:**

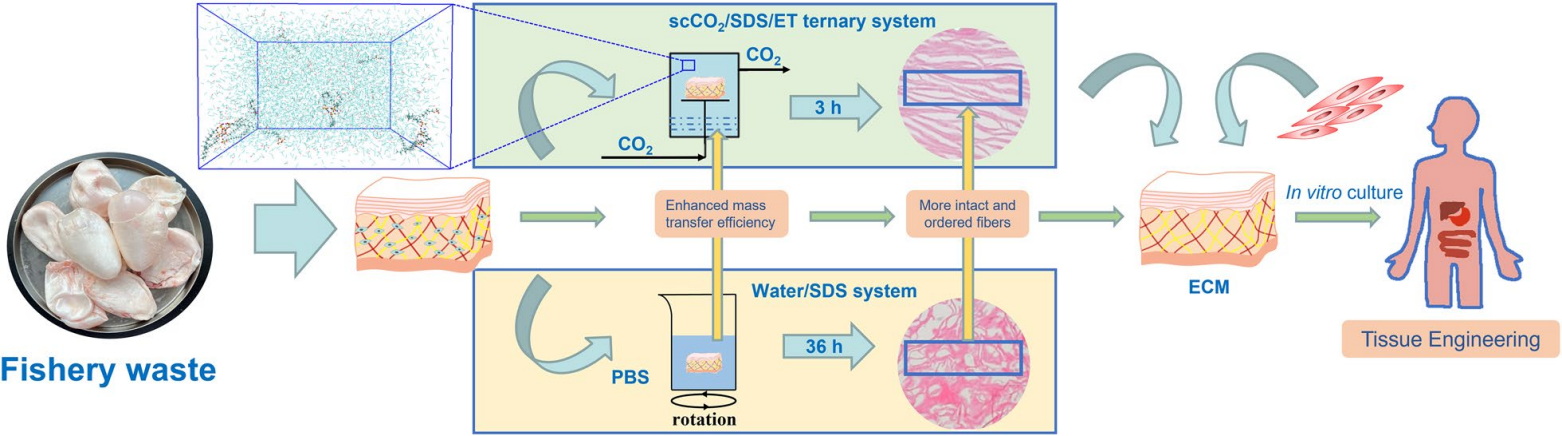

**Supplementary Information:**

The online version contains supplementary material available at 10.1186/s40643-022-00621-4.

## Introduction

Each year, 41,000 patients receive a transplant and 48,000 new patients are on waiting lists in Europe, while more than 100,000 patients are waiting for organs transplant in the United States (Duarte et al. 2022). The demand for organ transplantation is increasing rapidly all over the world, and the shortage of available organs has been the crucial limiting factor in clinical therapy for many patients. This situation urges researchers to explore new methods to achieve organ regeneration, such as direct replacement of damaged organs or regeneration of defective tissues through tissue engineering.

Extracellular matrix (ECM) is an extremely complex noncellular three-dimensional macromolecular network composed of multiple bioactive molecules secreted by cells, for instance, collagen, glycosaminoglycans, elastin, fibronectin, laminin, and several growth factors (Theocharis et al. 2016). ECM can deliver signals to cells and regulate various cell functions, such as growth, migration, and differentiation (Clause and Barker 2013; Frantz et al. 2010). Owing to its particular composition, natural microstructure and impact on both embryonic development and homeostasis of cells and organs, ECM can be an ideal biomaterial to respondent substrate to promote the regeneration of damaged tissues and an excellent scaffold for whole tissues and organs reparation. Decellularization is a method to obtain ECM from natural-derived tissues or organs by removing cellular components and antigens, by which the immunological danger of transplantation will be eliminated in principle. Several methods have been utilized to accomplish the decellularization procedure, for instance, chemical methods with acid (Gilbert et al. 2008), detergents (Chen et al. 2004; Hudson et al. 2004; Nakayama et al. 2010; Reing et al. 2010; Tudorache et al. 2007; Wainwright et al. 2010), alcohols (Montoya and McFetridge 2009), physical methods by adjusting temperature (Flynn 2010), pressure (Sawada et al. 2008) and electroporation (Sano et al. 2010), biological methods with enzymes (Price et al. 2010; Schenke-Layland et al. 2003) and chelating agents (Yang et al. 2009), or their combinations. Each method has been applied and presents unique advantages and inevitable limitations, depending on the type, density, structure, composition, and application of the tissues (Funamoto et al. 2010). Sodium dodecyl sulfate (SDS) is an anionic surfactant which has been a widely used chemical reagent in decellularization. SDS can lysis cell membranes, remove nuclear remnants and antigens more efficiently when compared with other detergents (Gilbert et al. 2006). However, as a kind of mutagen, the residual SDS has a negative effect on metabolic activity of endothelial cells (Cebotari et al. 2010) and is highly toxic to the recellularization process by changing cell genetic material and inducing cell mutation (Syed et al. 2014). Therefore, complete removing the residual SDS by washing process is necessary, but time consuming. The urinary bladder matrix prepared by SDS solution was washed with deionized (DI) water for 48 h and the DI water was changed every 12 ± 2 h (Kao et al. 2020), and even a 3-day washing processing was carried out to accomplish the removal of the detergent by phosphate buffered saline (PBS) washing (Liu et al. 2018). Obviously, excessive detergent exposure can destroy the connection between proteins and loosen fibers in the ECM (Gilpin and Yang 2017). Therefore, rapid removal of detergent from tissues after treatment should be a key point to maintain the structure of ECM during the decellularization procedure. Alizadeh et al. (2019) utilized the vacuum washing method with 12 h to promote the washing processing and obtain decellularized pericardium, which can not only remove the most of the SDS from the decellularized tissue, but also showed a better structure when compared with the PBS method. Therefore, selecting a suitable strategy that can enhance the mass transfer of SDS in the tissue, remove the residual SDS rapidly and reduce the contact time with tissue fibers is vital in decellularization.

Fish swim bladders (FSBs) used to be considered as garbage in fishery (Yadav et al. 2019), however, with the well-aligned natural collagen nano-fibrils, they have been applied in biomedical fields at present, such as the bio-piezoelectric nanogenerator used as a sensor (Ghosh and Mandal 2016). Meanwhile, after decellularization treatment, acellular fish swim bladders (AFSBs) with high content of collagen, excellent biocompatibility and biodegradable properties (Shahabipour et al. 2013; Yazdani Moghaddam et al. 2009), have been used as tissue engineering scaffolds in several fields (Bai et al. 2021; Li et al. 2021a, b; Sun et al. 2022). The outstanding advantage of scaffolds with AFSBs is that they can decrease the risk of foot and mouth disease, bovine spongiform encephalopathy and immune response when compared with mammalian-derived materials (Kim and Mendis 2006). Liu et al. (2020b) prepared AFSB-scaffolds crosslinked by glutaraldehyde (GA), which showed excellent anti-calcification properties against cardiovascular diseases. Li et al. (2019) utilized AFSBs as dura mater substitutes and the scaffolds can meet the requirements for stitches strength. However, as a dense connective tissue, FSBs require the decellularization medium with strong solvent effect and great dissolve ability to remove cellular components efficiently.

Supercritical carbon dioxide (scCO_2_) is the most intensively used supercritical fluid due to its chemical inertness, nonflammability, significant environmental advantages, and relatively mild critical conditions (*P*_c_ = 7.38 MPa, *T*_c_ = 31.1 °C; Beckman 2004). It can be easily obtained from the atmosphere with high purity and removed by depressurization without any toxic residues after processing. In the supercritical state, the diffusion coefficient, viscosity, and surface tension of scCO_2_ are similar to a gas, while the density is similar to a liquid (Sawada et al. 2008). The intermediate properties result in high mass transfer and permeability with excellent solvent strength at the same time, which makes supercritical fluid technology be successfully used in decellularization to efficiently remove the cellular debris and improve the efficacy by reducing the excessive decellularization time (Guler et al. 2017; Huang et al. 2017; Sawada et al. 2008; Seo et al. 2018; Sun et al. 2020; Wang et al. 2017). Moreover, decellularization can be utilized in combination with scCO_2_ and detergents, and maintain decellularization efficacy in relative low detergent concentrations (Duarte et al. 2022).

The ability of scCO_2_ extraction to remove cell components is linked to the solvent strength of supercritical fluid. However, as a nonpolar solvent, scCO_2_ has a low affinity for polar compounds (Yu et al. 2015), such as nucleic acids with hydrophobic cores and highly polar hydrophilic external backbones. To overcome the deficiency, improving operating conditions and adding entrainers have been proposed, of which ethanol is one of the most widely used cosolvents for the advantages of low toxicity, high polarity and superior miscibility with scCO_2_. ECMs from aorta (Guler et al. 2017; Sawada et al. 2008), adipose tissue (Wang et al. 2017), dermal matrix (Chou et al. 2020), cornea (Guler et al. 2017; Huang et al. 2017) and pericardium (Halfwerk et al. 2018) have been already obtained through scCO_2_ with ethanol. However, it is doubted whether the scCO_2_ strategy can be effective equally on dense connective tissues by inducing the cell lysis. The weak efficiency of scCO_2_ in permeating cell membranes may lead to incomplete cell removal. Thus, certain pretreatments and after treatments are utilized in decellularization process with scCO_2_ treatment to improve the decellular behavior. Casali et al. (2018) developed a hybrid strategy that the tissues were immersed in SDS aqueous as a cell lysis inducing pretreatment and followed a scCO_2_ processing with ethanol, which was successful in achieving qualified ECMs. Seo et al. (2018) washed the tissues with a mixture of antibiotics and DNase I for 5 days after scCO_2_ treatment to obtain acellular heart tissues. Moreover, adding polar compounds to enhance the solvating power of scCO_2_ is a potential method to accomplish tissue decellularization as well. Indeed, LS-54 was used as a cosolvent to decellularize high dense tissues by Antons et al. (2018), such as articular cartilage, tendon, and skin. A significant reduction of DNA was achieved in skin and tendon while not in cartilage. Sun et al. (2020) sought to accomplish a single-step decellularization and sterilization protocol for tendons processed by scCO_2_ with SDS and ETDA at 10.2 MPa and 39 °C for 2 h, but the results showed that the treated tendons with good mechanical properties still remained a significant percentage of cells. Nevertheless, scCO_2_ with polar modifiers is still a potential method for intensified decellularization. Notably, since the dissolution and dispersion of the entrainers in the supercritical fluid can affect the decellularization efficiency directly, it is critical to figure out the interactions between them. Molecular dynamic (MD) simulation is an useful tool to investigate the intermolecular interactions and behaviors in molecular level. Zhang et al. (2020) suggested that the retarded application of commercial surfactants is associated with their limited solubility in scCO2. Thus, they explored the mechanism of the increased solubility of surfactant sodium bis(2-ethylhexyl) sulfosuccinate in the CO_2_ using ethanol as cosolvent. Likewise, MD simulation was utilized in this study to explain the mechanism for the improved decellularization efficiency by a novel method.

In this study, we developed an efficient decellularization proposal utilizing the scCO_2_ processing with hybrid entrainers (SDS and ethanol) to prepare AFSBs with dense connective layer structure. The interactions among SDS, ethanol, and CO_2_ are expected to enhance the solubility of SDS in scCO_2_ and solvent capacity of the supercritical fluid, and the synergy between SDS and ethanol can lead to more thoroughly decellularization. Both of the hybrid cosolvents consisted of SDS and ethanol with scCO_2_ should be beneficial for decellularizing the dense structural FSBs. Based on the above concept, the decellularization experiments were carried out to harvest AFSBs using scCO_2_/SDS/ET ternary system, and the histological, mechanical properties, and the microstructure of matrixes after decellularization were characterized to certify the efficiency of the hybrid strategy. In addition, the intrinsic synergy mechanism between the hybrid additives in scCO_2_ was investigated through the molecular dynamic (MD) simulation.

## Materials and methods

### Materials

FSBs of chubs were purchased from a local aquatic product market (Shanghai, China). The surrounding fatty and connective tissues were removed with scissors and washed with PBS immediately. Then the tissues were cut into rectangles (5 cm × 2 cm) and stored at − 20 °C until treatment. CO_2_ (99.995%) was purchased from Air Liquide (Shanghai) Co., Ltd. SDS (AR, Macklin) was purchased from Shanghai Macklin Biochemical Co., Ltd. Ethanol (absolute, GENERAL-REAGENT), chloroform (AR, GENERAL-REAGENT) and other reagents were purchased from Shanghai Titan Scientific Co., Ltd., and used as received without further purification.

### Preparation of AFSBs

#### Standard decellularization with SDS

The standard SDS treatment on FSBs was followed the protocol as reported by Guler et al. (2017) Briefly, dissected FSBs were first immersed in SDS (1% w/v, 50 mL) for 12 h and washed with PBS for 24 h, the PBS was changed every 4 h. The fresh FSB without any treatment was defined as group Native, the FSB treated with SDS aqueous for 12 h and washed with PBS for 24 h was defined as group SDS-12-24. In addition, to explore the efficiency of SDS treatment and PBS washing processing, group with 1 h washing time was performed and defined as SDS-12-1.

#### Decellularization with scCO_2_

The FSB was placed on a porous stainless-steel holder to avoid contacting with entrainers directly and inserted into the vessel of a scCO_2_ extraction apparatus (Joel Hi-Tech Corporation (Dalian, China)) as shown in Additional file 1: Fig. S1, in which 10 mL SDS aqueous solution (5% w/v) and 30 mL ethanol (70% v/v) were placed at bottom as entrainers. The apparatus mainly consists of a pressurize pump, a heat exchanger, an extraction vessel and a separator. Liquid CO_2_ from the cylinder was compressed into the extraction vessel with a pressurizing pump until the pressure reached 25 MPa at 35 °C. Then the system was saturated statically for 30 min to allow the entrainers to dissolve in the scCO_2_ phase and penetrate into the tissues. Subsequently, the scCO_2_ system was operated for continuous extraction for 2 h to produce AFSBs. For the purpose of removing the trace amounts of residual SDS in the tissues, the AFSBs were washed by scCO_2_ extraction processing with ethanol alone as the entrainer for 1 h. The sample treated by scCO_2_ with SDS and ethanol was defined as ternary group scCO_2_/SDS/ET, while the comparative binary group scCO_2_/SDS and scCO_2_/ET were performed without ethanol and SDS, respectively.

### Histology

Histology analyses were performed to evaluate the decellularization efficiency and determine the structure of AFSBs before and after the treatment. In brief, tissues were fixed in 4% paraformaldehyde solution overnight at room temperature, then dehydrated through a serious of graded alcohol and embedded in paraffin. Subsequently, the paraffin-embedded tissues were cut into 5 μm thickness sections using a microtome (Leica, Germany) and deparaffinized in xylene for three times. The sections were stained with hematoxylin–eosin (H&E, Beyotime, China) or Verhoeff’s Van Gieson (EVG) Kit (Leagene, China) and then viewed by a light microscope (Nikon eclipse-Ti, Tokyo, Japan).

### DNA quantitation

To quantify the total DNA content, 20 mg of freeze-dried tissue was dissected into small pieces and digested with proteinase K at 56 °C thoroughly under agitation until no visible material remained. Then, the DNA was extracted from the solution via a classic protocol according to the manufacture’s protocol (TIANamp Genomic DNA Kit, China). The concentration of the DNA was determined by a NanoDrop instrument (NanoDrop 2000, Thermo Scientific, USA).

### Scanning electron microscopy (SEM)

To analyze the porous morphology and the fibrous ultrastructure of the tissues, SEM images were obtained from a JSM-6360LV (JEOL Ltd., Tokyo, Japan) scanning electron microscopy. In general, the samples were fixed in 4% paraformaldehyde solution at 4 °C and freeze-dried overnight. Then, the scanned fractured surfaces were sputtered-coated with platinum for 60 s at 30 mA. The section of each sample was scanned.

### Mechanical testing

Samples with a total length of 35 mm and a gauge length of 12 mm were prepared according to the GB/T 528-2009. Uniaxial tensile testing was conducted on a universal testing machine (AG-2000A, Shimadzu, Japan) at the test speed of 1 mm/min and increasing tension until failure. Tensile strength, strain, and elastic modulus (Young’s modulus, *E*′) were determined based on the apparent cross-sectional area of the “narrow bridge” sections. In particular, the *E*′ was determined by the slope of the second linear region of the tensile strength–strain curve (Liu et al. 2020a). Each decellularization condition was tested in triplicate.

### SDS quantitation

Residual SDS in the samples was quantified using the method proposed by Michael et al. (1992). The samples were cut into small pieces, homogenized and vortexed in deionized water for 24 h. After centrifuging the homogenate solution, 0.5 mL supernatant was mixed with an equal volume of methylene blue reagent and the mixture was extracted with 1 mL chloroform by thorough vortex. Subsequently, the methylene blue was extracted into the organic phase by centrifuging for 2 min. The organic phase was transferred into a 1.5 mL centrifugal tube which contained 100 mg anhydrous sodium sulfate and the tube was reversed several times to remove the water in the chloroform phase. The SDS concentration was correlated with the SDS content as well as the amount of tissue, while the amount of SDS was finally calculated by measuring the bottom organic phase at 600 nm with a microplate and comparing with a standard curve. In addition, to confirm that ethanol can enhance the solubility of SDS in scCO_2_ and the mass transfer rate of SDS in tissues, verification experiments were carried out. FSBs were immersed in SDS solution for 12 h and treated by scCO_2_ with or without ethanol for 1 h and defined as control groups SDS-12-scCO_2_/ET and SDS-12-scCO_2_. The SDS content of these two groups was detected as well.

### In vitro cell experiments

#### Cell proliferation

To evaluate the biocompatibility of the decellularized tissues, fibroblasts (L929) were inoculated onto the surface of the tissues to observe the proliferation. Thus, samples were cut into 8 mm × 8 mm squares and sterilized with ethanol (75% v/v) for 2 h and ultraviolet overnight before cell seeding, then placed at the bottom of a 48-well plate with stainless-steel rings (2 mm in height, 11 mm in outer diameter and 1 mm in thickness) upon them to prevent the samples from floating and cells leaking out. 10,000 L929 in 30 μL Dulbecco’s modified Eagle’s medium (DMEM) were pipetted on the top of the tissues and left to attach for 2 h in an incubator. Afterwards, the samples were immersed by adding 250 μL DEME and maintained a humid atmosphere of 95% air/5% CO_2_ (v/v) at 37 °C. The medium was changed every day and the CCK-8 assay was performed each two days. A 10% mixture of CCK-8 reagent in DEME was prepared and added to the wells for incubating 2 h. 100 μL supernatant of each well was measured at 450 nm using a microplate reader (SPECTRAmax384, Molecular Devices, USA).

#### Cytotoxicity

In order to detect the cytotoxic of the residual SDS in samples, CCK-8 tests were performed according to the manufacturer’s protocol. The samples were sterilized overnight by ultraviolet irradiation, and the extraction medium was prepared by immersing the samples in 500 μL DMEM for 24 h. Cells at the density of 3 000 cells/well were seeded in a 96-well plate. After cell adhesion, cell medium was replaced by extract medium and cultured for 1, 3, and 5 days. 10 μL CCK-8 was added into each well and incubated for 2 h, then the absorbance was measured at 450 nm using a microplate reader. OD_*S*_ represents the absorbance of the sample while OD_*B*_ represents the blank control group. The cell viability was calculated as follows: cell viability (%) = OD_*S*_/OD_*B*_ × 100%, *n* = 6.

### MD simulation details

To determine the synergy between SDS and ethanol as well as the enhancement mechanism of ethanol on the solubility and dispersion of SDS in scCO_2_, MD simulations were performed by GROMACS 5.0.7 (Abraham et al. 2015). The structures of SDS and ethanol molecules were optimized using B3LYP/6-11(G) method as shown in Additional file 1: Fig. S2 and CHARMM36 (Lee et al. 2014) force field parameters were obtained from SwissParam (Zoete et al. 2011). CO_2_ molecules were described by the three-site EPM2 model (Harris and Yung 1995), which can describe the physical and chemical properties of CO_2_ accurately in supercritical conditions. Initially, SDS molecules, CO_2_ molecules and ethanol molecules were loaded in a cubic box (8.0 × 8.0 × 10.0 nm^3^ without ethanol molecules or 8.0 × 8.0 × 12.0 nm^3^ with ethanol molecules; Zhang et al. 2020) by the PACKMOL software (Martínez et al. 2009). The periodic boundary condition was adopted in all directions. Different compositions of the simulation systems were summarized in Additional file 1: Table S1. In the system with ethanol, the fraction of ethanol was set to 10 molar% of CO_2_ (Dobbs et al. 1987).

Simulation details were presented as follows: The energy of the initial configuration was minimized using a steep integrator for 20,000 steps, a 100 ps canonical ensemble (NVT) was executed with the time step of 1 fs at 308.15 K, and then a 30 ns isothermal–isobaric ensemble (NPT) at 308.15 K and 25 MPa was carried out. In the end, the trajectories of last 10 ns MD simulation were used for data analysis. Herein, an accurate leap-frog stochastic dynamics integrator was used for all the simulations, the Berendsen thermostat with relaxation time of 1 ps was employed to control the temperature at 308.15 K and pressure at 25 MPa. Furthermore, the particle mesh Ewald method (PME) was applied to treat the long-range electrostatic interactions. A 6–12 Lennard–Jones interaction potential was used to describe the van der Waals interactions, where the coulomb interaction was set at 1.2 nm. After the simulations, all the snapshots were displayed with the VMD software (Humphrey et al. 1996).

### Statistical analysis

The results are expressed as mean values ± standard deviation (SD) for DNA, SDS quantification, cell proliferation and cytotoxicity. The differences between group Native, scCO_2_/SDS/ET, scCO_2_/ET, scCO_2_/SDS and SDS-12-24 were compared by an unpaired, two-tailed *t* test. *p* ≤ 0.05 was considered statistically significant.

## Results and discussion

### Biological characterization

#### Biological staining

**Fig. 1:**
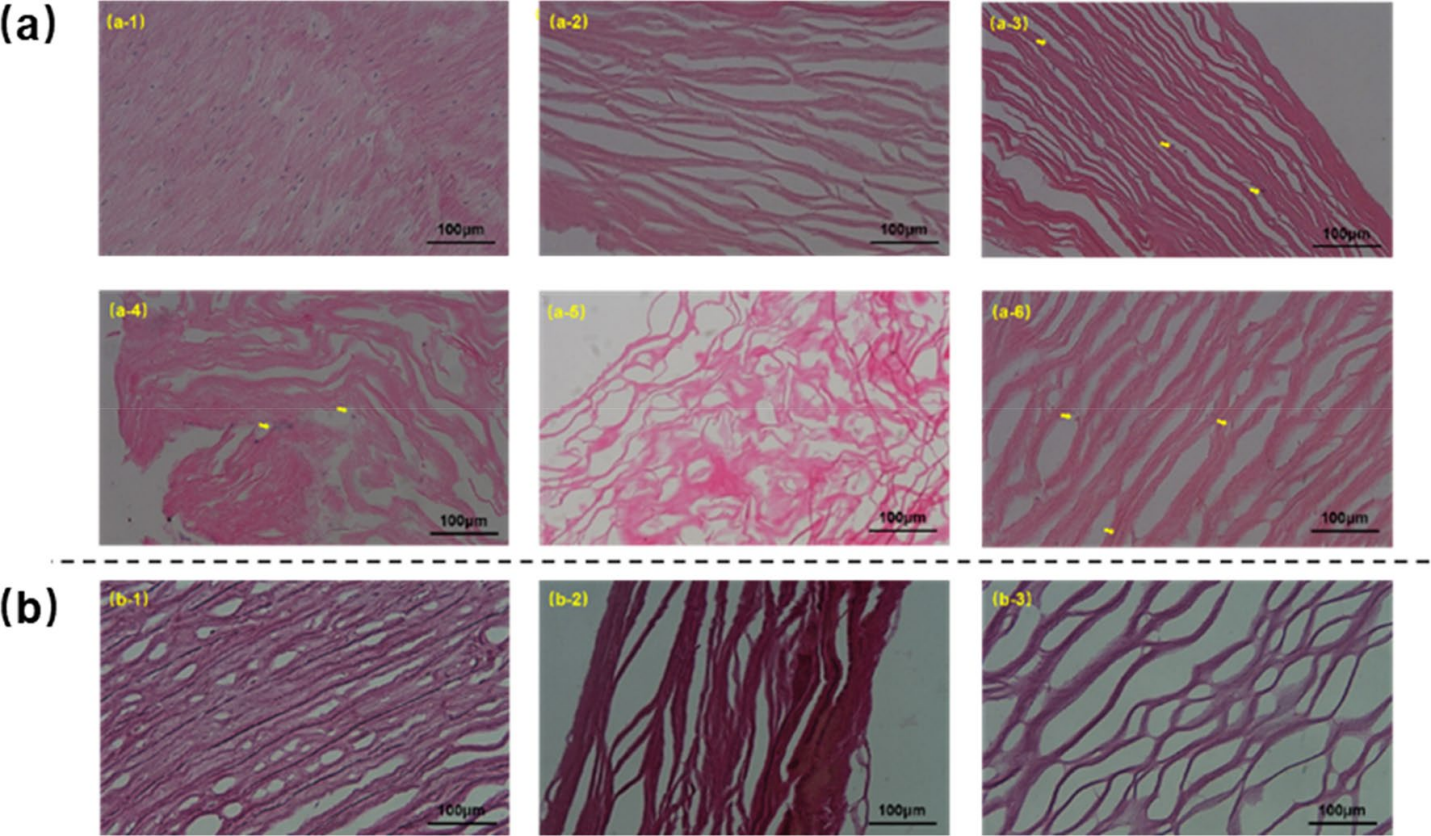
Histology of AFSBs specimens: H&E staining sections of (**a-1**) Native, (**a-2**) scCO_2_/SDS/ET, (**a-3**) scCO_2_/ET, (**a-4**) scCO_2_/SDS, (**a-5**) SDS-12-24 and (**a-6**) SDS-12-1. EVG stained sections of (**b-1**) Native, (**b-2**) scCO_2_/SDS/ET and (**b-3**) SDS-12-24

In order to assess the extent of decellularization, H&E staining of AFSBs proposed by Crapo et al. (2011) was utilized to detect nuclear material in tissues, shown in Fig. 1. In group Native, intact oval nuclei are stained blue-purple by hematoxylin which can be observed readily, while the proteins are bound to eosin and present pink. Dark areas of cell debris pointed with yellow arrows can be observed in group scCO_2_/SDS and scCO_2_/ET, showing incomplete cell removal. Since ethanol may not have sufficient counteractive solvent strength to remove cell debris completely (Antons et al. 2018), the binary scCO_2_ system with ethanol alone may not be efficient enough to remove cell debris from tissues. Differently, the weak decellularization efficiency of scCO_2_/SDS system should be ascribed to the poor solubility of SDS in scCO_2_.

Meaningfully, there is no visible cellular materials can be found after decellularization treatment in group scCO_2_/SDS/ET and SDS-12-24 shown in Fig. 1a, indicating that both two methods can accomplish complete decellularization. It is not surprising to observe the broader fiber gaps in all AFSBs as compared to group Native. The dehydration leads to fibrous adhesions and stiffness while the extraction and washing by different operations with hybrid entrainers is able to extract components in the ECM which connect to the tissues not tightly enough. It should be noted that fibers in AFSBs of ternary group scCO_2_/SDS/ET are more ordered and intact in a comparison with group SDS-12-24 owing to the transient contact time with SDS. The disordered and confused morphology in group SDS-12-24 demonstrates that SDS has severe damage to ECM of AFSBs as compared to the combination of scCO_2_, SDS, and ethanol. Differ from individual entrainer, the synergy between hybrid entrainer of SDS and ethanol enable better results, both in efficiency of cell removal and retention of tissue structures. In order to maintain the structure of the AFSBs for comparison, group SDS-12-1 using unitary SDS solution was performed with the same washing time as ternary group scCO_2_/SDS/ET, and the fibers morphology after treatment indeed become more intact and neater with lower solvent effect while some cell debris are clearly remained. It means that, though SDS is capable of disrupting cell membranes and isolating DNA components, the SDS aqueous solution cannot achieve decellularization thoroughly and retain ECM structures at the same time. That is because the contact time of SDS with ECM is too long at relative high concentration in tissue with low removal rate of SDS with PBS solution. It directly leads to the disruption of ECM structures. Therefore, reducing the contact time between SDS and tissues allows for a retention of integral fibers, while rapid removal of SDS is the crucial to maintain the ultrastructure of AFSBs.

EVG staining was also carried out to investigate the arrangement and properties of elastin fibers in AFSBs, in which elastin fibers are stained as black and collagen fibers are stained as red. As shown in Fig. 1b, the collagen fibers in group Native are abundant and uniform as well as the elastin fibers are regular and in a parallel orientation. In ternary group scCO_2_/SDS/ET, collagen fibers become uneven due to the dehydration of the tissues during the extraction. Although disappeared in some areas due to the stacking between the collagen fibers which hide the elastin fibers, elastin fibers can be observed clearly. Notably, scCO_2_ combined with ethanol can evidently accelerate the decellularization process with SDS through enhancing the mass transfer rate both into and out of tissue, because scCO_2_ has low viscosity, zero surface tension, and strong dissolve and wash cell debris ability assisted with ethanol. Furthermore, the damage to ECM of AFSBs is reduced by removing the SDS in a short time through scCO_2_ extraction. The synergy between SDS and ethanol with scCO_2_ should be attribute to the increased interaction in ternary group scCO_2_/SDS/ET, which also has been verified by the following MD simulation. Conversely, the damage to ECM of group SDS-12-24 with long treatment time is confirmed by significant gaps between fibers, widespread breakage and disappeared elastin fibers. Besides the structure analysis of AFSBs, the fragile mesh of fibers can lead to a weak mechanical property, which will be discussed later.

#### DNA quantification

**Fig. 2:**
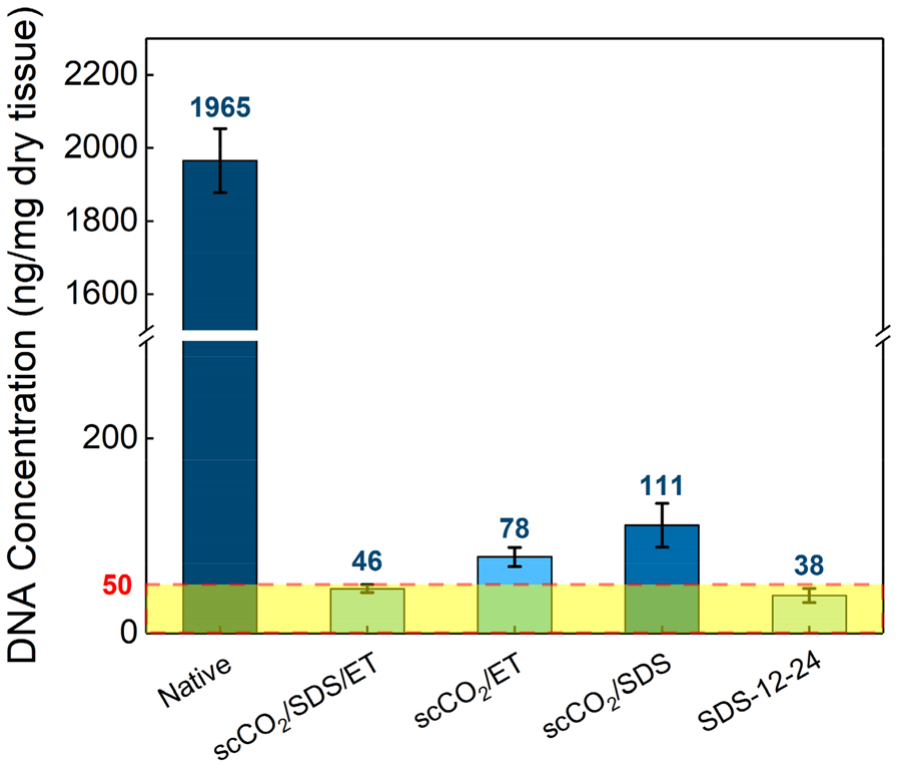
DNA content of AFSBs. Values below 50 represent complete decellularization

Another criterion of decellularization is the total amount of double-stranded DNA in extracellular matrix. It is considered to be decellularized completely that the residual double-stranded DNA value in ECM should be less than 50 ng/mg dry tissue (Crapo et al. 2011). In our work, the DNA content of AFSBs for each treatment group is shown in Fig. 2. It is clear to see that group SDS-12-24 has the minimal residual DNA content of 38.46 ng/mg dry tissue, a reduction from 1965.29 ng/mg in native FSBs, which sacrifices the structure of the tissues and the efficiency of decellularization. DNA content of AFSBs using binary system scCO_2_/ET and scCO_2_/SDS are also dramatically decreased to 78.24 and 110.8 ng/mg, respectively. However, the corresponding AFSBs are not decellularized completely. Only using ternary system scCO_2_/SDS/ET, AFSBs can achieve the qualified DNA content, which is 45.6 ng/mg. It also indicates that the ternary system with hybrid entrainers is more efficient to achieve the decellularization standard than the binary system. It is worth noting that the sensitivity limitations of the DNA quantify due to the low optical density near the decellularization threshold and the tolerance of the quantification device (Casali et al. 2018). The results of DNA quantification and H&E staining both can certify the efficiency of the synergy between scCO_2_ and hybrid entrainers in decellularization and maintaining the ultrastructure.

### Mechanical and structural characterization

#### Scanning electron microscopy

**Fig. 3:**
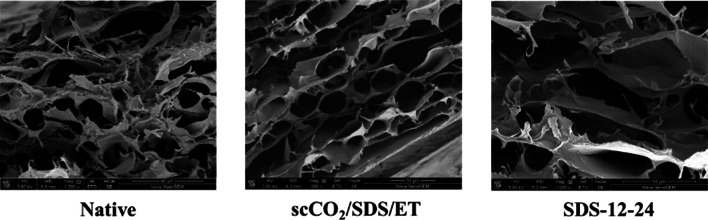
SEM images of Native, scCO_2_/SDS/ET and SDS-12-24

In order to explore the ultrastructure of AFSBs treated by different decellularization strategies, the morphology analysis was performed by SEM testing on the cross-sections of ECM. As shown in Fig. 3, the fresh FSBs present stratification of fiber bundles and interconnected porous structures, of which the pores are intact and distributed uniformly. It can be seen that the pores remain intact and evenly distributed after treating by ternary system scCO_2_/SDS/ET, in the meantime, the pore walls are smoother and thinner with no residual fibrous debris due to the removal of weak bonding components through supercritical extraction. However, after complete decellularization in group SDS-12-24, the stratification is more obvious, the pore walls are damaged and the pores become incomplete. This result correlates with the destruction of the fragile fibrous structure between the layers in long-term exposure to SDS solution. Furthermore, it should be noted that the damage in the middle of the SDS-12-24 structure is more severe than that at the edge owing to the low diffusion rate and accumulation of SDS.

#### Mechanical testing

**Fig. 4:**
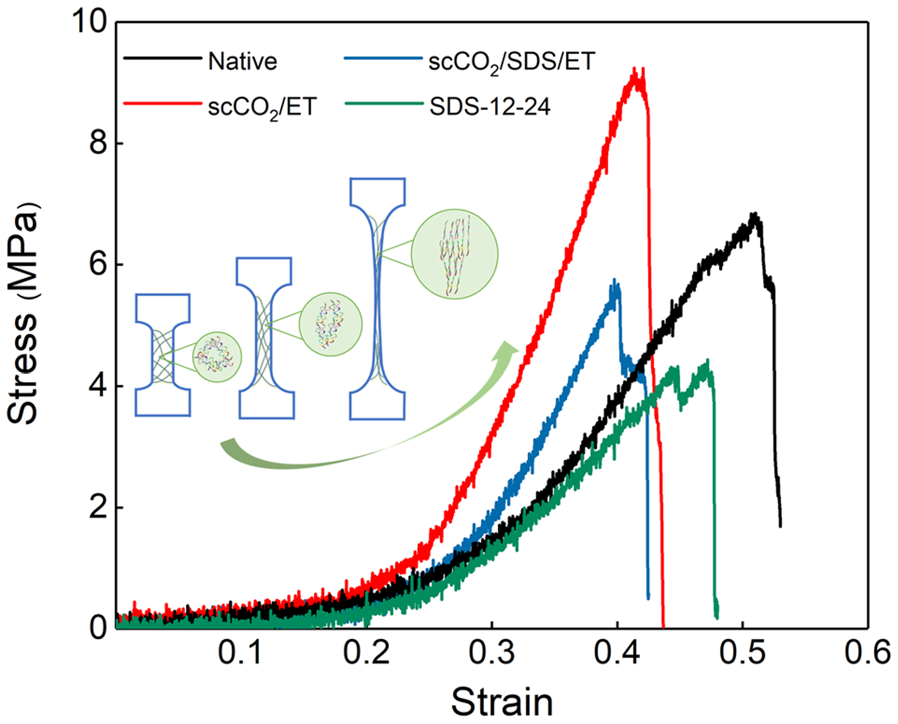
Stress–strain curves from different treatment of Native, scCO_2_/SDS/ET, scCO_2_/ET and SDS-12-24

Sufficient mechanical properties are vital for ECM in clinical application, as the matrix might be subjected to suturing and stretching forces during surgery. The native tissue FSBs has high anisotropy, and both the tensile strength and elastic module in circumferential direction are higher than those in radial direction (Liu et al. 2020b). As the stretching progresses, the fibrous meshes begin to distort, the fibers are subjected to the tension and the partial collagen chains may be exposed to the tension at the extreme stretching, while the response of collagen can be influenced by the internal structure and inhomogeneity throughout its length (Kirkness et al. 2019). Thus, the tensile strength in circumferential direction of AFSBs was tested to analyze the mechanical performance in this work, and the results are shown in Fig. 4. Correspondingly, the *E*′ values have been calculated as the slope of the stress–strain curves in the linear region and listed in Table 1. In Fig. 4, all strain–stress curves start with a sharp linear region and show the same triangular shape as pericardial tissues (Choe et al. 2018; Marinval et al. 2018). When compared with the group Native, the tensile strength of group SDS-12-24 significantly reduces from 6.82 to 4.21 MPa as a result of the destruction of the internal ultrastructure and fibers. However, the tensile strength of group scCO_2_/ET increases to 9.04 MPa and the elongation at break decreases severely, which is due to the severe tissue dehydration during scCO_2_ decellularization with ethanol (Halfwerk et al. 2018; Sawada et al. 2008). For the ternary system of scCO_2_/SDS/ET, the tensile strength and elongation at break of sample are 5.61 MPa and 40.10%, respectively. The water introduced into supercritical extraction via SDS aqueous in scCO_2_/SDS/ET treatment can reduce dehydration and stiffness of tissues, however, more ECM components can be extracted out, leading to lower tensile strength when compared with group scCO_2_/ET. Notably, *E*′ is associated with the distribution and orientation of fiber bundles (Halfwerk et al. 2018). The *E*′ value of group scCO_2_/SDS/ET is higher than that of group SDS-12-24, suggesting that decellularization treatment assisted with scCO_2_ and ethanol can maintain the mechanical properties better than SDS aqueous.

**Table 1:** Mechanical property data of native, scCO_2_/SDS/ET, scCO_2_/ET, and SDS-12-24

Sample	Elastic modulus (MPa)	Tensile strength (MPa)	Elongation at break (%)
Native	31.18	6.82	51.05
scCO_2_/SDS/ET	39.99	5.61	40.10
scCO_2_/ET	55.41	9.04	41.50
SDS-12-24	20.36	4.21	44.51

### SDS quantification

**Fig. 5:**
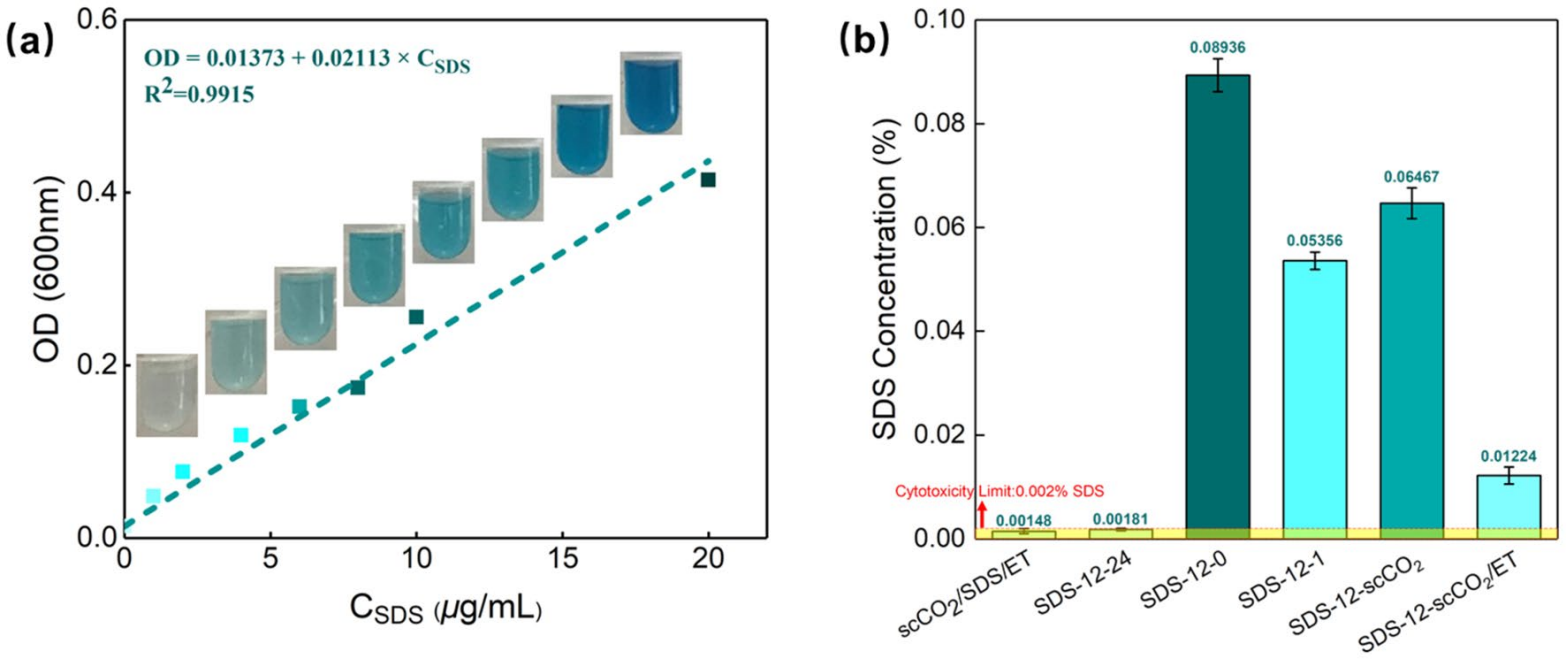
Quantitation of residual SDS: **a** SDS standard absorbance curve and **b** residual SDS in samples

As mentioned before, the retention of structures is closely concerned with the rapidly removal of SDS and the substantial SDS remained in the ECM will disrupt the fibers structure like sample SDS-12-24. In addition, the concentration of SDS more than 0.002% is cytotoxic which can affect cell viability and recellularization during tissue regeneration (Zvarova et al. 2016). Thus, the relative SDS standard absorbance curve was obtained by measuring a series of standard SDS graded solutions and the residual SDS contents in AFSBs were correspondingly quantified, which are shown in Fig. 5, respectively. It can be found that only the treatment with scCO_2_/SDS/ET and SDS-12-24 can remove SDS below the toxicity threshold at the same level, reaching 0.00148% and 0.0018%, respectively. That is same to the result of DNA quantification. However, the washing treatment for group scCO_2_/SDS/ET can takes less time when compared with only PBS for SDS-12-24 to achieve the same decellularization effect, in particular, the efficiency of scCO_2_/ET for SDS removal will become more favorable with the tissue thickness increasing.

To further verify that scCO_2_ washing with ethanol can increase the removal of SDS, residual SDS in group SDS-12-0, SDS-12-1, SDS-12-scCO_2_, and SDS-12-scCO_2_/ET were quantified. It can be seen that the residual SDS concentration of group SDS-12-scCO_2_ decreases from 0.0894 to 0.0647%, which is higher than that of group SDS-12-1, indicating that pure scCO_2_ has a weak effect on SDS removal. At the same time, the introduction of ethanol to scCO_2_ can significantly decreases the residual SDS concentration to 0.0122%. The efficiency of SDS removal is associated with its solubility in the washing fluid and the mass transfer rate of the washing fluids. In this novel decellularization strategy, ethanol can enhance the solubility of SDS in scCO_2_, thus improving the efficiency of SDS removal.

### Biocompatibility

#### Cell proliferation

**Fig. 6:**
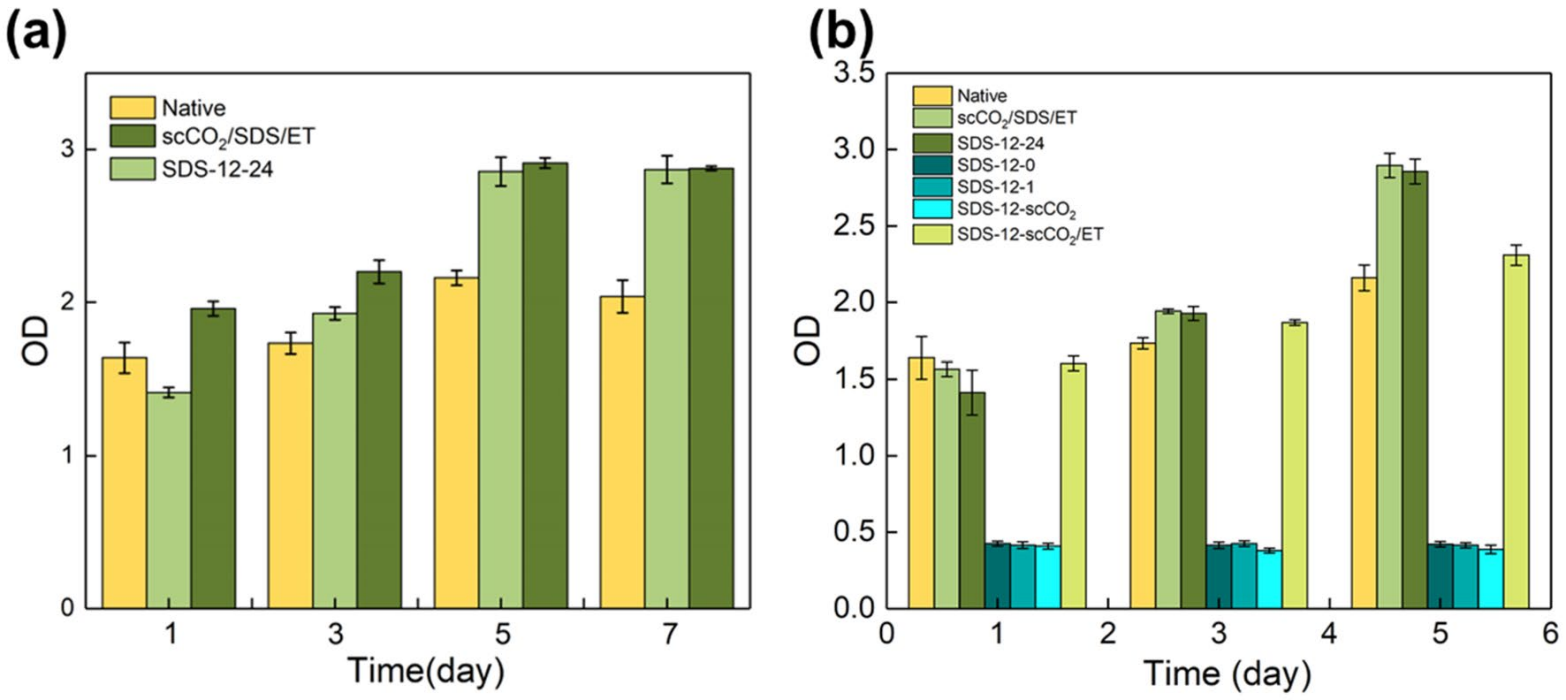
CCK-8 assay results of **a** cell proliferation on the AFSBs (based 8 mm × 8 mm) on 1, 3, 5, and 7 days, **b** cell cytotoxicity on 1, 3, and 5 days. The Native group was served as the control group for comparison. All values are the means with ± SD. *n* = 3 (*p < 0.05)

CCK-8 assay was performed to confirm the biocompatible of AFSBs and explore the proliferation of cells, and the results are shown in Fig. 6. For the first 5 days, both the experimental groups and the control group show cell proliferation, indicating the cells are active. Meanwhile, the cell number in experiment groups is higher than control group due to the rougher surfaces of AFSBs which can promote the adhesion and proliferation of cells. The rougher surfaces are the results of the decline in cellular components and dehydration after decellularization. On the 7th day, the cell number leveled off since all surfaces have been covered with cells, limiting cell growth. From the results, it appears that the AFSBs obtained by scCO_2_/SDS/ET and SDS-12-24 treatment are biocompatible, nontoxic, and suitable for the growth of cells.

#### Cytotoxicity

CCK-8 assay was carried out to evidence the toxicity tissues with residual SDS as well. There is no change among the OD values of group SDS-12-0, SDS-12-1, and SDS-12-scCO_2_ as the excess SDS remained which has been confirmed through SDS quantitation is cytotoxic. Cells in SDS-12-scCO_2_/ET grow slowly due to the inhibition of trace amount of SDS in tissues, though it is not fatal. Cytotoxicity test results are consistent with SDS quantitative results, which can further confirm the efficiency of scCO_2_ treatment with hybrid entrainer in removing SDS and maintaining the biocompatibility of AFSBs.

### Simulation results

Because the ethanol has a favorable interaction and compatibility with scCO_2_ and can improve the micellar behavior of SDS (Plastinin et al. 2020), it may be a suitable cosolvent to modify the supercritical fluids (Frolov and Kiselev 2014). In this work, MD simulation was utilized to explore the effect of ethanol on the dispersion of SDS in scCO_2_. The configurations of the dynamic equilibrium simulation systems are shown in Additional file 1: Fig. S3. It can be observed that the SDS molecules trend to gather together rather than dispersing in scCO_2_ without ethanol, and the size of the cluster increases with the number of SDS molecules increasing. The hydrophilic head groups of SDS are clustered in the interior, while the hydrophobic tails extend outwards to contact with CO_2_. Oppositely, the SDS clusters disappear and the molecules disperse more uniformly in the presence of ethanol.

**Fig. 7:**
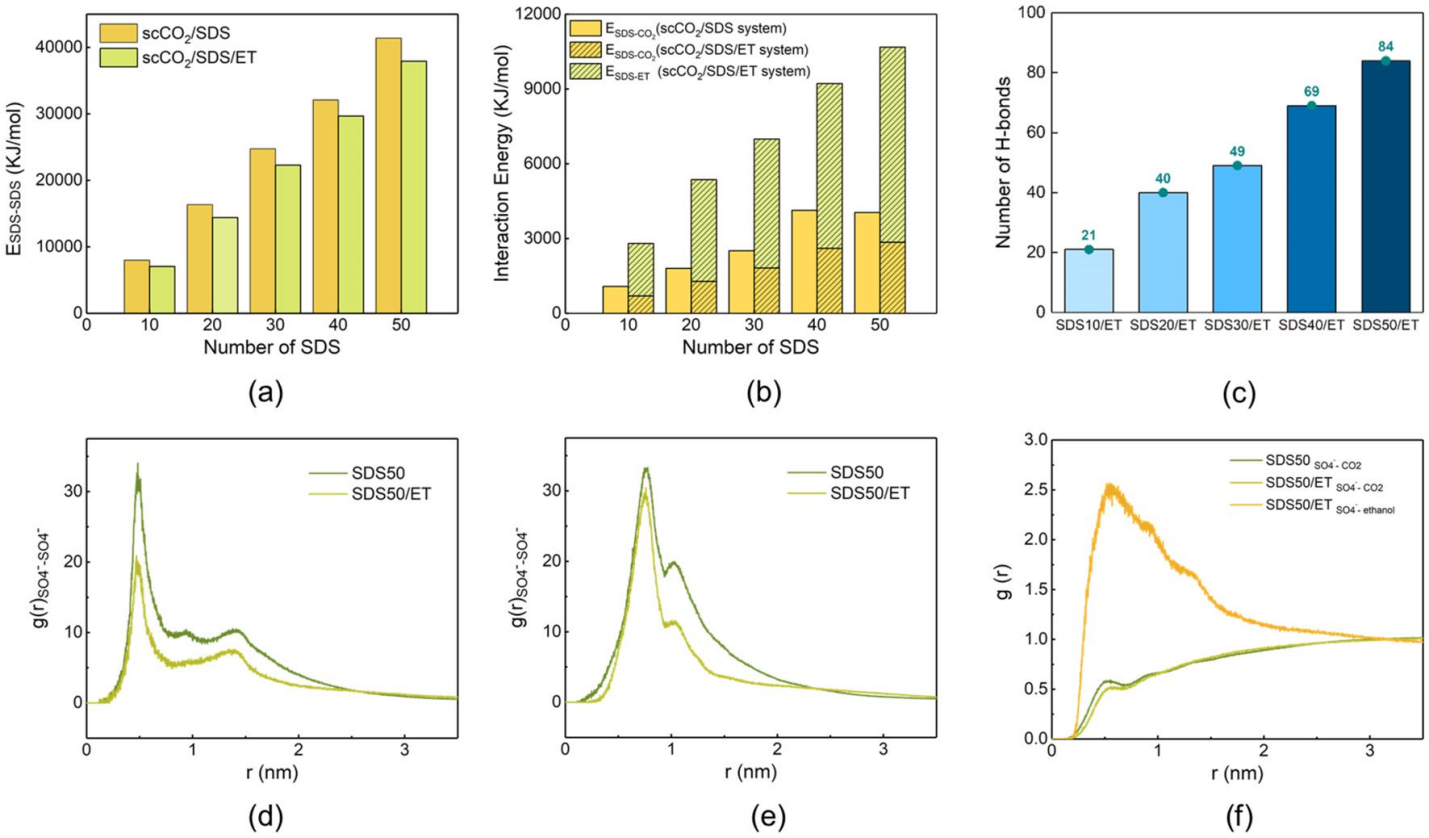
**a** *E*_SDS-SDS_, **b** *E*_SDS-CO2_, and *E*_SDS-ET_ in scCO_2_/SDS and scCO_2_/SDS/ET systems (kJ/mol), **c** number of H-bonds between SDS and ethanol molecules, RDF between pairs of **d** SO_4_^−^–SO_4_^−^, **e** SO_4_^−^–Na^+^ and **f** SO_4_^−^–CO_2_ and SO_4_^−^–ethanol in SDS50 and SDS50/ET systems

The interaction energies between SDS and SDS (*E*_SDS-SDS_), SDS and CO_2_ (*E*_SDS-CO2_) as well as SDS and ethanol (*E*_SDS-ET_) were calculated comprehensively to investigate the interaction among SDS, ethanol, and CO_2_ molecules quantitatively and shown in Fig. 7a, b. The addition of ethanol can significantly reduce the interaction energy between SDS molecules in the case of the same number of SDS molecules, resulting in the dissolution of SDS clusters at the macro-scale. Meanwhile, it is not surprising to find that the values of *E*_SDS-SDS_ and *E*_SDS-CO2_ increase with the increasing numbers of SDS molecules. The SDS50 and SDS50/ET systems are utilized as examples, the value of *E*_SDS-SDS_ reduces from 41,403.63 kJ/mol in the SDS50 system to 37,905.93 kJ/mol in the SDS50/ET system. However, the value of *E*_SDS-CO2_ in SDS50/ET system decreases compared with SDS50, and the decreased value is more than compensated by the interaction energy between SDS and ethanol. Generally speaking, nonpolar tails of SDS molecules can interact with CO_2_ molecules and the polar heads which are turned out to interact with alcohol molecules (Chaschin et al. 2020). Ethanol can reduce the solute self-interaction and improve the solute–solvent attraction forces to enhance the dispersion of SDS in scCO_2_.

The interaction between SDS and ethanol is attributed to the formation of hydrogen bonds (H-bonds) between O atom in SO_4_^−^ groups of SDS and H atom in ethanol. To verify the assumption, H-bonds between SO_4_^−^ groups and ethanol molecules were calculated in all systems. As shown in Fig. 7c, there are indeed H-bonds between SO_4_^−^ groups and ethanol molecules and the number of H-bonds increases with the increasing number of SDS molecules.

To further investigate the variation of interactions in the supercritical system, the radial distribution functions (RDF) between different components were calculated. RDF analysis can reflect the micro-structural characteristics trend of pair–pair interactions in the system. It describes the probability of finding A at the distance of r from B, as shown in Eq. (1):

1 $$g_{{\text{AB}}} \left( {\text{r}} \right) \, = \frac{1}{{\rho_{{\text{AB}}} \cdot 4\pi 2\cdot \Delta r}}\frac{{\sum_{J = 1}^{N_{{\text{AB}}} } \Delta N_{{\text{AB}}} \left( {r \to r + \Delta r} \right)}}{{N_{{\text{AB}}} }}$$

where *g*_AB_(*r*) is the intensity of RDF, the subscripts A and B represent two types of atoms or groups, *ρ*_AB_ is the system bulk density, *N*_AB_ presents the number of pairs of A and B, *∆N*_AB_ is the pair number of A (or B) with a distance interval from *r* to *r* + ∆*r* around B (or A).

Figure 7d–f shows several types of RDF between different pairs in typical systems of SDS50 and SDS50/ET. In Fig. 7d, e, both the intensities of *g*(*r*) SO_4_^−^–SO_4_^−^ and *g*(*r*) SO_4_^−^–Na^+^ in SDS50/ET system are lower than those in SDS50 system, indicating the better dispersion of SDS molecules after introducing ethanol. In addition, no obvious peak can be found in *g*(*r*) SO_4_^−^–CO_2_ in Fig. 7f, implying the weak interaction between sulfuric acid groups and CO_2_ in each system. Furthermore, the probability of finding ethanol near the SO_4_^−^ groups is much higher than that of CO_2_ and the intensity of *g*(*r*) SO_4_^−^–ethanol is high, showing the relatively stronger interaction between ethanol and sulfuric acid groups. The results are highly consistent with the interaction energies characteristics. All of the above evidence proposes clearly that ethanol can interact with the SO_4_^−^ groups of SDS to promote the affinity and dispersion of SDS with scCO_2_.

## Conclusions

In this work, an effective noncytotoxic decellularization strategy has been proposed using SDS ternary system with scCO_2_ as the green extraction fluid and ethanol as the cosolvent to prepare AFSBs. When compared with unitary SDS aqueous and binary system scCO_2_/SDS and scCO_2_/ET, the ternary system scCO_2_/SDS/ET is more effective in decellularization and removal of SDS due to the enhanced solubility of SDS in supercritical fluids with ethanol and the high mass transfer rate. AFSBs treated with the novel strategy avoids exposure to SDS prolonged, which can maintain intact fibers and eliminate the toxicity of tissues, resulting in promoted mechanical properties and biocompatibility. In the ternary scCO_2_/SDS/ET system, SDS is a vital component to improve the solvent strength of supercritical extraction fluid and disrupt the interaction between cell components and ECM, while ethanol acts as a cosolvent to promote solubility of SDS in scCO_2_ and increases the polarity of the system. The synergy among SDS, ethanol, and scCO_2_ in ternary system has been confirmed by calculating the interaction energies, RDF and H-bonds using MD simulation. The polar cosolvent, ethanol, can interact with the hydrophilic head groups of SDS to enhance the interaction between SDS and supercritical fluid, which improved the solubility and dispersion of SDS in scCO_2_. The hybrid entrainers can enhance the solvent strength of supercritical fluid and achieve more efficient decellularization when compared with individual entrainer. Therefore, the findings in this work can provide new approaches for the preparation of ECMs with excellent mechanical properties and biocompatibility of different tissues using hybrid entrainers with scCO_2_ in decellularization.

### Supplementary Information

**Additional file 1:** Additional information of the supercritical extraction system. **Table S1**. Different compositions of scCO2/SDS/ET systems in MD simulations. **Figure S1. ** Schematic diagram of the scCO2 extraction apparatus. **Figure S2.** Molecular structures of (a) CO2 (b) Ethanol (c). **Figure S3.** Snapshots for final configurations in of different simulated systems SDS.

## Data Availability

The datasets used and/or analyzed during the current study are available from the corresponding author on reasonable request.

## Abbreviations

ECM: Extracellular matrix
FSBs: Fish swim bladders
AFSBs: Acellular fish swim bladders
GA: Glutaraldehyde
SDS: Sodium dodecyl sulfate
DI water: Deionized water
PBS: Phosphate buffered saline
scCO_2_: Supercritical carbon dioxide
ET: Ethanol
MD simulation: Molecular dynamic simulation
CO_2_: Carbon dioxide
H&E: Hematoxylin–eosin
SEM: Scanning electron microscopy
*E′*: Young’s modulus
L929: Fibroblasts
DMEM: Dulbecco’s modified Eagle’s medium
NVT: Canonical ensemble
NPT: Isothermal−isobaric ensemble
PME: Particle mesh Ewald method
RDF: Radial distribution functions
