# Supplementary material for: Preparation of extracellular matrix of fish swim bladders by decellularization with supercritical carbon dioxide
Source: Bioresour Bioprocess. 2023 Feb 21;10(1):14. doi: 10.1186/s40643-022-00621-4 (PMC10991867; doi:10.1186/s40643-022-00621-4)
Supplement: Supplementary file 1 — Additional file 1: Additional information of the supercritical extraction system. Table S1. Different compositions of scCO2/SDS/ET systems in MD simulations. Figure S1. Schematic diagram of the scCO2 extraction apparatus. Figure S2. Molecular structures of (a) CO2 (b) Ethanol (c). Figure S3. Snapshots for final configurations in of different simulated systems SDS. [file 40643_2022_621_MOESM1_ESM.docx]

**Additional file 1**

# Preparation of extracellular matrix of fish swim bladders by decellularization with supercritical carbon dioxide

Yuqing Han^a^, Bingyan Zhang^a^, Jinjin Li^a^, Lian Cen^b^, Ling Zhao^a,b^, Zhenhao Xi^a,b,*^

^a^ State Key Laboratory of Chemical Engineering, School of Chemical Engineering, East China University of Science and Technology, Shanghai 200237, China

^b^ Shanghai Key Laboratory of Multiphase Materials Chemical Engineering, East China University of Science and Technology, Shanghai 200237, China

*** Corresponding author**

Prof. Zhenhao Xi

Email address: [zhhxi@ecust.edu.cn](mailto:zhhxi@ecust.edu.cn)

**Table 1 supplementary. Different compositions of scCO_2_/SDS/ET systems in MD simulations**

| **Case** | **System** | **Number of CO_2_** | **Number of SDS** | **Number of ethanol** |
| --- | --- | --- | --- | --- |
| 1 | SDS10 | 6000 | 10 |  |
| 2 | SDS10/ET | 6000 | 10 | 640 |
| 3 | SDS20 | 6000 | 15 |  |
| 4 | SDS20/ET | 6000 | 15 | 640 |
| 5 | SDS30 | 6000 | 30 |  |
| 6 | SDS30/ET | 6000 | 30 | 640 |
| 7 | SDS40 | 6000 | 40 |  |
| 8 | SDS40/ET | 6000 | 40 | 640 |
| 9 | SDS50 | 6000 | 50 |  |
| 10 | SDS50/ET | 6000 | 50 | 640 |


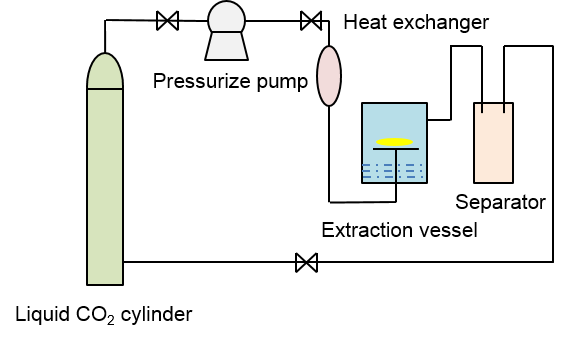


**Figure 1 supplementary. Schematic diagram of the scCO_2_ extraction apparatus**


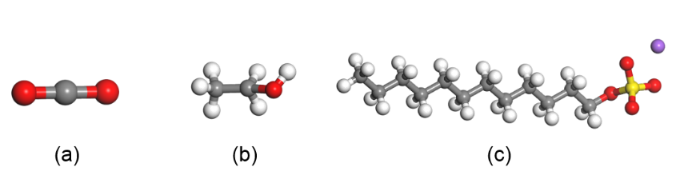


Figure 2 supplementary. Molecular structures of (a) CO_2_ (b) Ethanol (c) SDS


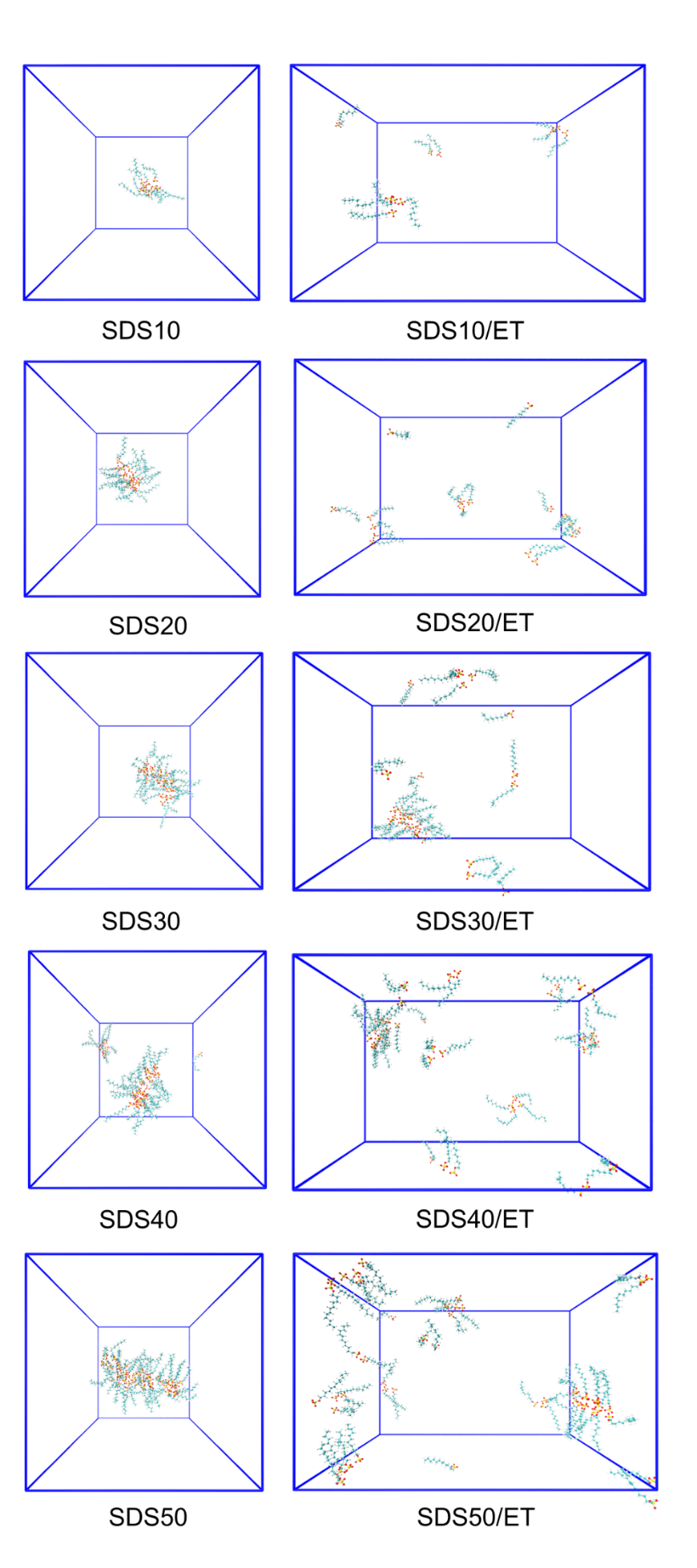


**Figure 3 supplementary. Snapshots for final configurations in of different simulated systems**
